# Two Novel Proline-Containing Catechin Glucoside from Water-Soluble Extract of *Codonopsis pilosula*

**DOI:** 10.3390/molecules23010180

**Published:** 2018-01-16

**Authors:** Fu-Ying Qin, Li-Zhi Cheng, Yong-Ming Yan, Bao-Hua Liu, Yong-Xian Cheng

**Affiliations:** 1 State Key Laboratory of Phytochemistry and Plant Resources in West China, Kunming Institute of Botany, Chinese Academy of Sciences, Kunming 650201, China; qinfuying@mail.kib.ac.cn; 2 School of Pharmacy, Henan University of Chinese Medicine, Zhengzhou 450008, China; 3 University of Chinese Academy of Sciences, Beijing 100049, China; 4 Guangdong Key Laboratory for Genome Stability & Disease Prevention, School of Pharmaceutical Sciences, School of Medicine, Shenzhen University Health Science Center, Shenzhen 518060, China; 13424039397@163.com (L.-Z.C.); yanym@szu.edu.cn (Y.-M.Y.); ppliew@szu.edu.cn (B.-H.L.)

**Keywords:** *Codonopsis pilosula*, Catechin glucosides, Choushenflavonoids A and B, Proline, SIRT1

## Abstract

Choushenflavonoids A (**1**) and B (**2**), two unusual proline-containing catechin glucosides, were isolated from the roots of *Codonopsis pilosula* cultivated in a high-altitude location of Yunnan province. Their structures were determined by spectroscopic data and chemical methods. Specifically, the absolute configuration of glucose residue in **1** and **2** was assigned by acid hydrolysis followed by derivatization and gas chromatography (GC) analysis. In addition, biological evaluation of **1** and **2** against Sirtuin 1 (SIRT1) was carried out.

## 1. Introduction

The genus *Codonopsis* is a perennial herb in the family Campanulaceae, and contains 42 species predominantly found in Central, East, and South Asia. There are approximately 40 species that can be found in China and these are mainly distributed in the western and northern parts of China, such as Gansu, Sichuan, Guizhou, Shaanxi and Shanxi provinces [1,2]. The dry roots of *Codonopsis pilosula*, known as Dang Shen in Chinese, are a common traditional medicine that has been used for thousands of years [3]. It has been used to replenish Qi, nourish blood, strengthen immunity, improve appetite, decrease blood pressure, etc. [4,5]. Previous phytochemical studies on this plant revealed that it contains phytosteroids, sesquiterpenes, triterpenes, alkaloids, alkylalcohol glycosides, phenylpropanoid glycosides, polyacetylene glycosides, neolignan, and polysaccharides [6,7]. Additionally, modern pharmacological investigations have shown that the extract of *C. pilosula* could regulate immunity, improve learning and memory ability, inhibit iNOS, and so forth [8,9]. During our study on traditional Chinese medicines, *C. pilosula*, produced in Yunnan province, was investigated, this herb is mainly distributed at 2800–4200 m slopes of grassland and shrub and is known locally as Chou Shen in Yunnan province. It has been used as a food in Yunnan province, and can be seen in vegetable markets every autumn or winter. Secondary metabolites are influenced by the environment; to gain insight into the difference of *C. pilosula* in different regions, we undertook a study on Chou Shen grown at high altitude in Yunnan province, which resulted in the isolation of two unusual proline-containing catechin glucosides. In this paper, we describe their isolation, structural identification and biological activity against SIRT1, a nicotinamide adenosine dinucleotide (NAD)-dependent deacetylase.

## 2. Results and Discussion

### 2.1. Structure Elucidation of the Compounds

**Figure 1:**
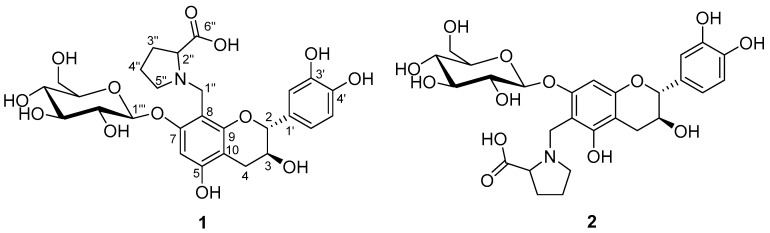
The structures of **1** and **2** from *Codonopsis pilosula.*

The EtOH extract of *C. pilosula* was suspended in water and partitioned with EtOAc. The aqueous layer was concentrated and submitted to a combination of chromatography to afford compounds **1** and **2** (Figure 1).

**Figure 2:**
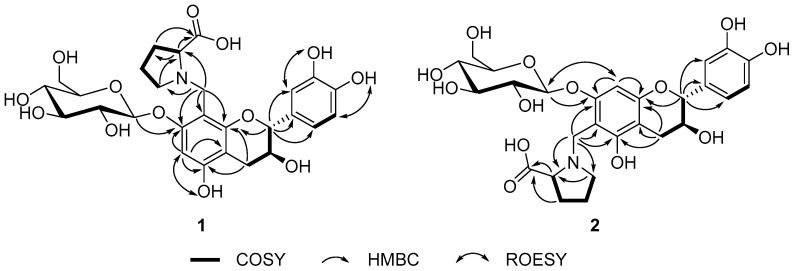
Key COSY, HMBC and ROESY correlations for **1** and **2**.

Choushenflavonoid A (**1**), isolated as a brownish yellow powder, has the molecular formula C_27_H_33_NO_13_ (12 degrees of unsaturation), based on analysis of its HRESIMS at *m*/*z* 602.1846 [M + Na]^+^ (C_27_H_33_NNaO_13_, calcd. for 602.1850), ^13^C-NMR, and DEPT spectra (Figure S6). The ^1^H-NMR spectrum (Table 1) exhibited signals at *δ*_H_ 4.77 (d, *J* = 6.4 Hz, H-2), 3.92 (m, H-3), 2.59 (dd, *J* = 16.3, 4.7 Hz, Ha-4), and 2.45 (dd, *J* = 16.3, 7.1 Hz, Hb-4) and the presence of an ABX system [*δ*_H_ 6.75 (brs, H-2′), 6.70 (d, *J* = 8.1 Hz, H-5′), 6.62 (brd, *J* = 8.1 Hz, H-6′)]. The ^13^C-NMR and DEPT spectra (Table 1) showed that this substance contains 27 carbons, including six methylene (one oxygenated), twelve methine (four sp^2^, eight sp^3^), and nine quaternary carbons (all sp^2^ including five oxygenated and one carboxyl). In addition, the observation of one anomeric proton at *δ*_H_ 4.79 (d, *J* = 7.2 Hz), five methine (*δ*_C_ 101.1, 77.2, 76.2, 72.8, 69.2), and one methylene (*δ*_C_ 60.4) in the ^1^H- and ^13^C-NMR spectra of **1** revealed the presence of a sugar residue. These data are similar to those for catechin-7-*O*-β-d-glucoside [10]. Apart from one isolated methylene (*δ*_H_ 4.20, d, *J* = 13.1 Hz; *δ*_H_ 4.12, d, *J* = 13.1 Hz), the three unassigned methylene (*δ*_C_ 52.2, 28.0, 23.1), one methine (*δ*_C_ 66.6), and one carboxyl group (*δ*_C_ 169.9) might be a proline residue if taken into consideration of the presence of a nitrogen atom and unsaturation degrees required by the molecular formula. This conclusion was confirmed by analysis of the ^1^H-^1^H COSY correlations of H-2′′/H-3′′/H-4′′/H-5′′ (Figure S9 and Figure 2) as well as the HMBC (Figure S8) correlations of H-2′′/C-6′′, H-5′′/C-2′′ (Figure S8, and Figure 2). Judging from the chemical shift of C-1′′, the isolated methylene group (CH_2_-1′′) should be connected to a nitrogen atom, which was supported by the observation of the HMBC correlation of H-1′′/C-2′′, C-5′′. The HMBC correlations of H-1′′/C-7, C-8, C-9, H-1′′′/C-7 finally determined the planar structure of **1** as shown. The *trans* relationship of H-2/H-3 was readily concluded from the coupling constant of H-2 (d, *J* = 6.4 Hz). For the configuration of sugar moiety, acid hydrolysis of **1** followed by TLC comparison and GC analysis allowed the assignment of d-glucose. In detail, the l-cysteine methyl ester hydrochloride derivatives of the hydrolysis product of **1**, d-, and l-glucose were prepared and subjected to GC comparison. The retention time (Figures S1–S3) for that of **1** is 21.319 min, close to that of d-glucose (21.280 min), rather than l-glucose (21.696 min), clarifying the type of sugar and its configuration. The presence of a proline residue in **1** made it structurally intriguing. It is rather challengeable to assign the absolute configuration at C-2′′, whereas, we could tentatively conclude that the proline residue should be l-form, since l-rather than d-configuration is more abundant or common in nature. Taken together, the structure of **1** was identified and named as choushenflavonoid A.

Choushenflavonoid B (**2**), isolated as a brownish yellow powder, has the molecular formula C_27_H_33_NO_13_ (12 degrees of unsaturation), based on analysis of its HRESIMS *m*/*z* 602.1857 [M + Na]^+^ (C_27_H_33_NNaO_13_ calcd. for 602.1850), ^13^C-NMR, and DEPT spectra (Figure S14). The ^13^C-NMR and DEPT spectra (Table 1) showed that this substance contains 27 carbons, including six methylene (one oxygenated), twelve methine (four sp^2^, eight sp^3^), and nine quaternary carbons (all sp^2^ including five oxygenated and one carboxyl). The NMR spectral data of **2** are very similar to those of **1**, differing in that C-1′′ in **2** was connected to C-6, which was identified by the HMBC correlations of H-1′′/C-5, C-6, C-7. Additionally, the HMBC correlations (Figure S16 and Figure 2) of H-1′′′/C-7 clearly indicated the location of the sugar moiety. In this way, the planar structure of **2** was deduced. Similarly, the *J*_H-2,H-3_ (6.8 Hz) value indicated a *trans* relationship. Evidence for the presence of a d-glucose residue in the structure of **2** came from analysis of the acid hydrolysis product in the manner as described for **1**. Thus, the structure of **2** was determined to be that shown in Figure 1.

### 2.2. Biological Evaluation

SIRT1, as a nicotinamide adenosine dinucleotide (NAD)-dependent deacetylase, is reported to have biological functions affecting various processes, such as aging, cancer, metabolism, neurodegeneration, and immunity [11,12,13]. Inhibition of SIRT1 expression leads to increased tumor cell death and no toxic effect on normal cells in culture inhibitor [14]. With this assay in hand, Compounds **1** and **2** were tested for their inhibitory activity against SIRT1. Unfortunately, both **1** and **2** were found not to be active in this assay, even at a concentration of 200 μM (data not shown).

## 3. Experimental Section

### 3.1. General Procedures

Column chromatography was undertaken on D101 macroporous resin (Tianjin Haiguang Chemical Co., Ltd., Tianjing, China), RP-18 (40–60 µm; Daiso Co., Tokyo, Japan), and Sephadex LH-20 (Amersham Pharmacia, Uppsala, Sweden). Optical rotations were collected on a Horiba SEPA-300 polarimeter (Horiba, Kyoto, Japan). UV spectra were obtained on a Shimadzu UV-2401PC spectrometer (Shimadzu Corporation, Tokyo, Japan). CD spectra were measured on a Chirascan instrument (Applied Photophysics, Surrey, UK). GC analysis was performed using an Agilent 6890N gas chromatography instrument (Agilent Technologies, Santa Clara, CA, USA). Semi-preparative or analytic HPLC was carried out using an Agilent 1200 liquid chromatograph (Agilent Technologies, Santa Clara, CA, USA) the column used was a 250 mm × 9.4 mm, i.d., 5 µm. NMR spectra were recorded on an AV-600 spectrometer (Bruker, Karlsruhe, Germany) with TMS as an internal standard. ESIMS and HRESIMS were collected by an Agilent G6230TOF MS spectrometer (Agilent Technologies, Santa Clara, CA, USA).

### 3.2. Plant Material

The roots of *C. pilosula* were collected from Xundian County in Yunnan province, China, a cultivation base, in November, 2015. The cultivated plant was ever identified by Prof. De-Yuan Hong at Beijing Institute of Botany, Chinese Academy of Sciences, and a voucher specimen (1016268) was deposited at the Herbarium of Kunming Institute of Botany, Chinese Academy of Sciences, China.

### 3.3. Extraction and Isolation

The dried roots of *C. pilosula* (20 kg) were powdered and soaked by 85% aqueous EtOH (4 × 80 L × 24 h) to give a crude extract, which was suspended in water followed by extraction with EtOAc to afford an EtOAc-soluble extract and an aqueous extract. The latter was divided into three parts (Fr.1–Fr.3) by using a D101 macroporous resin column eluted with gradient aqueous EtOH (5%, 40%, and 100%). Fr.2 (30 g) was separated by Sephadex (Amersham Pharmacia, Uppsala, Sweden) LH-20 (MeOH) to yield two fractions (Fr.2.1 and Fr.2.2). Fr.2.2 (19 g) was separated by a RP-18 column (MeOH/H_2_O, 10–70%) to get eight fractions (Fr.2.2.1–Fr.2.2.8). Of these, Fr.2.2.2 (2.3 g) was purified by Sephadex LH-20 (MeOH), followed by semi-preparative HPLC (MeCN/H_2_O, 8%, flow rate: 3 mL/min) to yield compound **1** (3.5 mg, R_t_ = 8.2 min) and (MeOH/H_2_O, 15%, flow rate: 2.5 mL/min) to yield compound **2** (5.2 mg, R_t_ = 28.5 min).

### 3.4. Compound Characterization Data

*Choushenflavonoid **A***(**1**): Brownish yellow powders; [α]_D_^21.9^ –42.4 (*c* 0.15, MeOH); UV (MeOH) *λ*_max_ (logε) 285 (3.18), 211 (4.13) nm; ESIMS *m*/*z* 602 [M + Na]^+^, HRESIMS *m*/*z* 602.1846 [M + Na]^+^ (calcd. for C_27_H_33_NNaO_13_, 602.1850); ^1^H- and ^13^C-NMR data, see Table 1.

*Choushenflavonoid **B***(**2**): Brownish yellow powders; [α]_D_^24.3^–42.7 (*c* 0.12, MeOH); UV (MeOH) *λ*_max_ (logε) 286 (3.16), 211 (4.08) nm; ESIMS *m*/*z* 602 [M + Na]^+^, HRESIMS *m*/*z* 602.1857 [M + Na]^+^ (calcd. for C_27_H_33_NNaO_13_, 602.1850); ^1^H- and ^13^C-NMR data, see Table 1.

### 3.5. Acid Hydrolysis and Sugar Analysis

A solution of **1** or **2** (2.0 mg) in 1 N HCl was stirred at 70 °C for 5 h. After cooling, the mixtures were extracted with EtOAc. The aqueous layer was neutralized with 1 N NaOH and concentrated in vacuo, which was subsequently dissolved in anhydrous pyridine (2 mL). To these solutions, l-cysteine methyl ester hydrochloride (2.0 mg) was added, and the mixtures were stirred at 60 °C for 1 h and concentrated in vacuo at 0 °C. Slow addition of 1-(trimethylsiyl) imidazole to the mixtures was followed by stirring at 60 °C for 1 h. Aliquots (4 µL) of the supernatants were subjected to chiral GC analysis to determine that d-glucose units present in **1** and **2** [15].

### 3.6. SIRT1 Inhibition

For examination of the SIRT1 inhibition of the compounds, each well contained 0.5 U of SIRT1 enzyme, 1000 μM of NAD^+^ (Enzo Life Sciences, New York, NY, USA), 100 μM of SIRT1 peptide substrate (Enzo Life Sciences, New York, NY, USA), and SIRT1 assay buffer (50 mM Tris-HCl, pH 8.0, 137 mM NaCl, 2.7 mM KCl, 1 mM MgCl_2_, 1 mg/mL BSA) along with the test compounds at an indicated concentration. The plate was incubated at 37 °C for 30 min and the reaction was stopped using Fluor de Lys developer II solution (Enzo Life Sciences, New York, NY, USA) containing 2 mM nicotinamide. The plate was further incubated at 37 °C for another 30 min and the samples were read by a fluorimeter with an excitation wavelength of 360 nm and emission wavelength of 460 nm [16].

## 4. Conclusions

To conclude, this study led to the isolation of two unusual proline-containing catechin glucosides, making **1** and **2** novel natural product hybrids. To the best of our knowledge, these are the first examples of flavonoids with a flavanol skeleton containing a proline moiety.

## Figures and Tables

**Table 1:** ^1^H- (600 MHz) and ^13^C-NMR (150 MHz) data of **1** and **2** in DMSO-*d*_6_ (*δ* in ppm, *J* in Hz).

Position	*δ*_H_	*δ*_C_		*δ*_H_	*δ*_C_
2	4.77, d (6.4)	81.3 d	2	4.59, d (6.8)	81.0 d
3	3.92, m	65.5 d	3	3.88, m	65.8 d
4	2.59, dd (16.3, 4.7)	26.7 t	4	2.70, dd (16.2, 4.9)	27.6 t
	2.45, dd (16.3, 7.1)			2.46, dd (16.2, 7.2)	
5		157.6 s	5		156.1 s
6	6.34, s	94.8 d	6		101.3 s
7		155.6 s	7		155.5 s
8		97.3 s	8	6.18, s	94.4 d
9		154.0 s	9		155.3 s
10		102.3 s	10		102.8 s
1′		130.0 s	1′		130.2 s
2′	6.75, br. s	114.1 d	2′	6.69, br. s	114.4 d
3′		144.9 s	3′		146.3 s
4′		145.0 s	4′		146.4 s
5′	6.70, d (8.1)	115.3 d	5′	6.68, d (8.1)	115.1 d
6′	6.62, br. d (8.1)	117.5 d	6′	6.58, br. d (8.1)	118.2 d
1′′	4.20, d (13.1)	45.0 t	1′′	4.13, d (13.2)	47.5 t
	4.12, d (13.1)			4.08, d (13.2)	
2′′	3.80, dd (9.3, 5.2)	66.6 d	2′′	3.63, (overlap)	67.1 d
3′′	2.08, m	28.0 t	3′′	2.17, m	28.3 d
	1.89, m			1.86, m	
4′′	1.82, m	23.1 d	4′′	1.86, m	20.8 d
	1.68, m			1.68, m	
5′′	3.30, overlap	52.2 t	5′′	3.25, overlap	53.0 t
	2.97, m			2.87, m	
6′′		169.9 s	6′′		171.3 s
1′′′	4.79, d (7.2)	101.0 d	1′′′	4.79, d (7.9)	101.1 d
2′′′	3.28, overlap	72.8 d	2′′′	3.24, overlap	73.1 d
3′′′	3.25, overlap	76.2 d	3′′′	3.24, overlap	76.4 d
4′′′	3.21, overlap	69.2 d	4′′′	3.13, m	69.6 d
5′′′	3.21, overlap	77.2 d	5′′′	3.24, overlap	77.0 d
6′′′	3.66, d (11.7)	60.4 t	6′′′	3.64, d (11.7)	60.6 t
	3.52, dd (11.7, 3.1)			3.42, dd (11.7, 5.7)	
5-OH	9.91, s				
3′-OH	9.08, s				
4′-OH	8.86, s				

## Supplementary Materials

The following are available online.
