# Supplementary material for: Two Novel Proline-Containing Catechin Glucoside from Water-Soluble Extract of Codonopsis pilosula
Source: Molecules. 2018 Jan 16;23(1):180. doi: 10.3390/molecules23010180 (PMC6017075; doi:10.3390/molecules23010180)
Supplement: Supplementary file 1 [file molecules-23-00180-s001.pdf]

Supplementary Materials: Two new proline-containing catechin glucosides from water soluble extract of *Codonopsis pilosula*

Fu-Ying Qin, Li-Zhi Cheng, Yong-Ming Yan, Bao-Hua Liu and Yong-Xian Cheng*

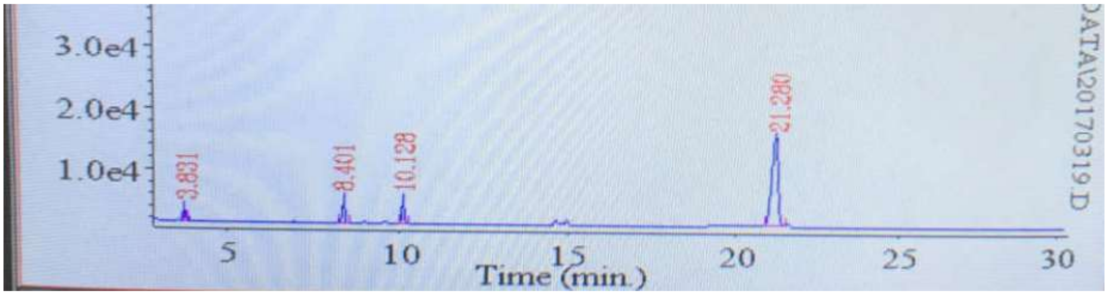

Figure S1. GC analysis of the derivative of D-glucose

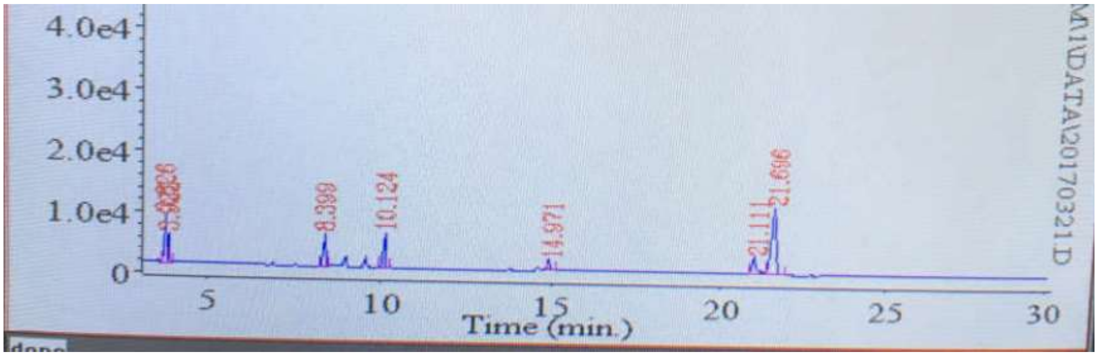

Figure S2. GC analysis of the derivative of L-glucose

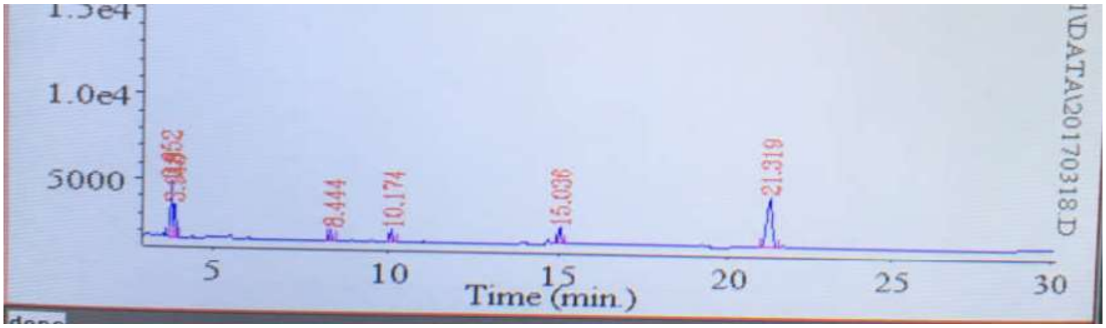

Figure S3. GC analysis of the derivative of compound **1** after hydrolysis

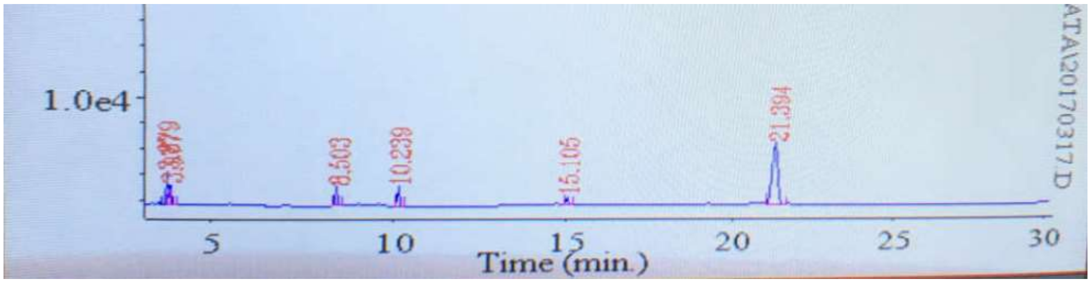

Figure S4. GC analysis of the derivative of compound **2** after hydrolysis

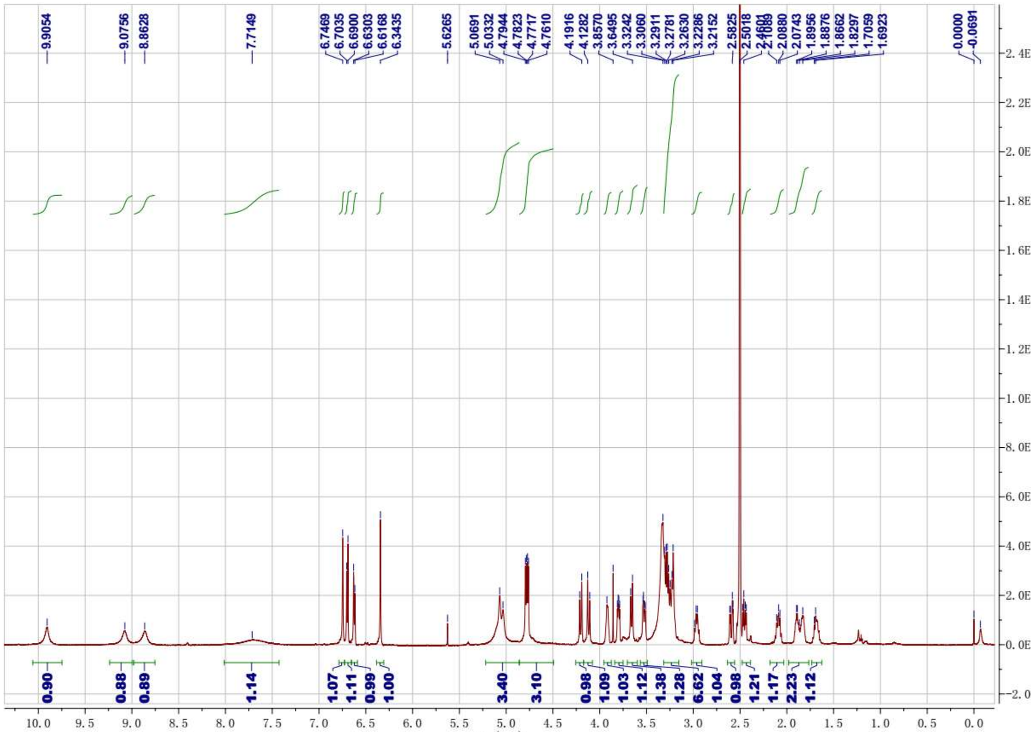

Figure S5.^{1}H NMR spectrum of **1** in DMSO-d_6

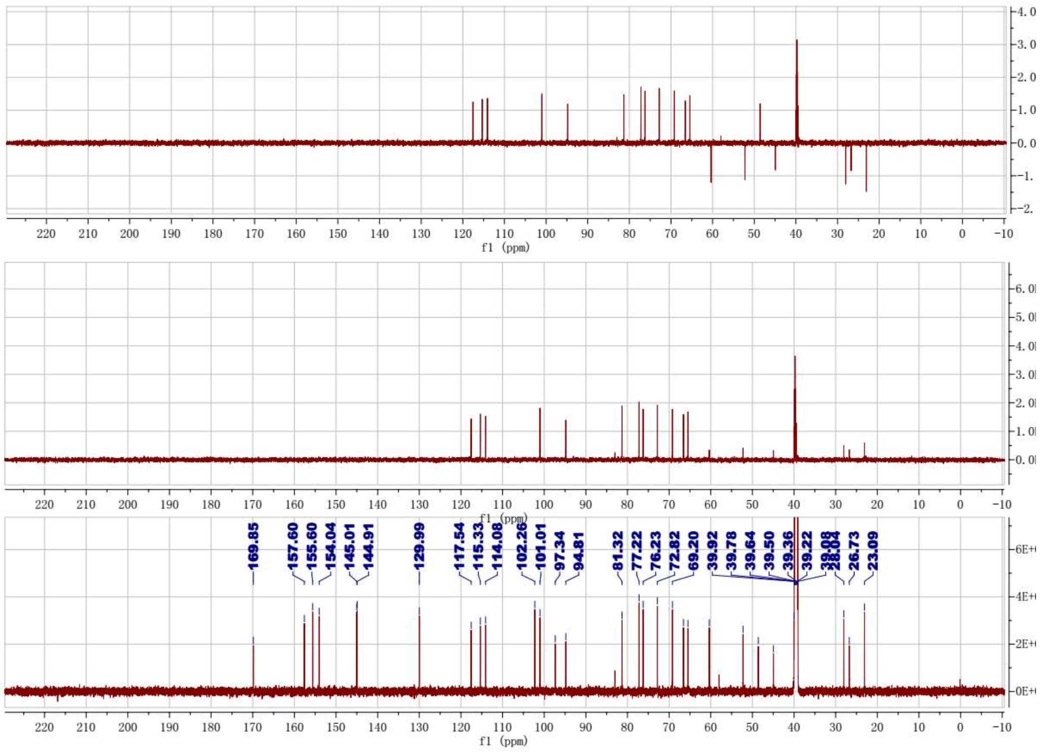

Figure S6. ^{13}C NMR and DEPT spectra of **1** in DMSO-d_6

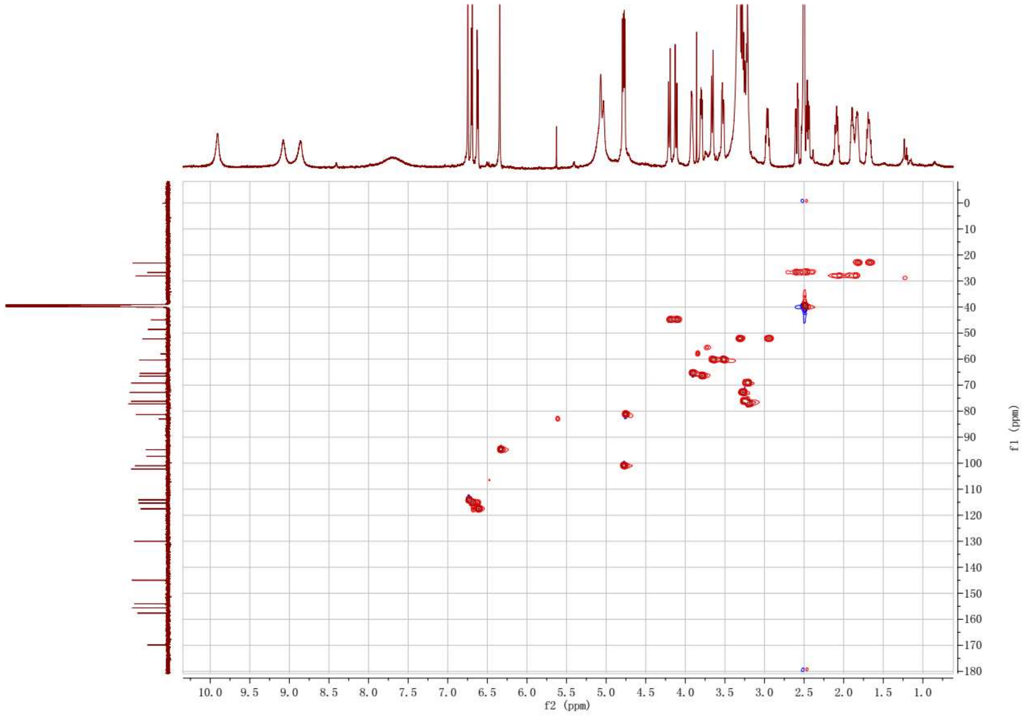

Figure S7. HSQC spectrum of **1** in DMSO-d_6

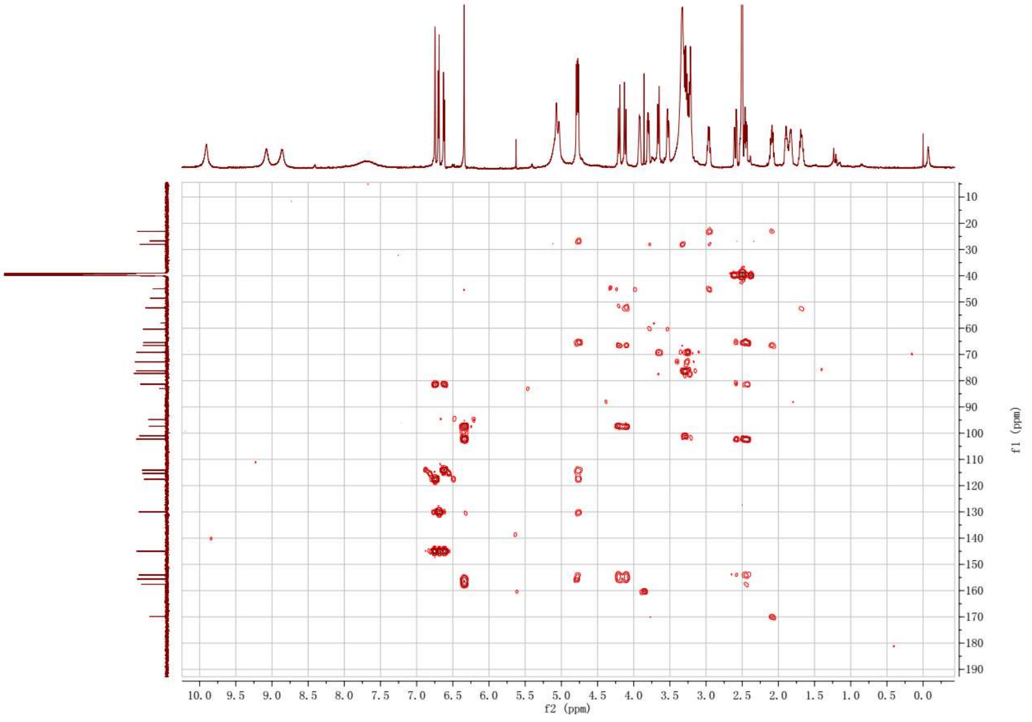

Figure S8. HMBC spectrum of **1** in DMSO-d_6

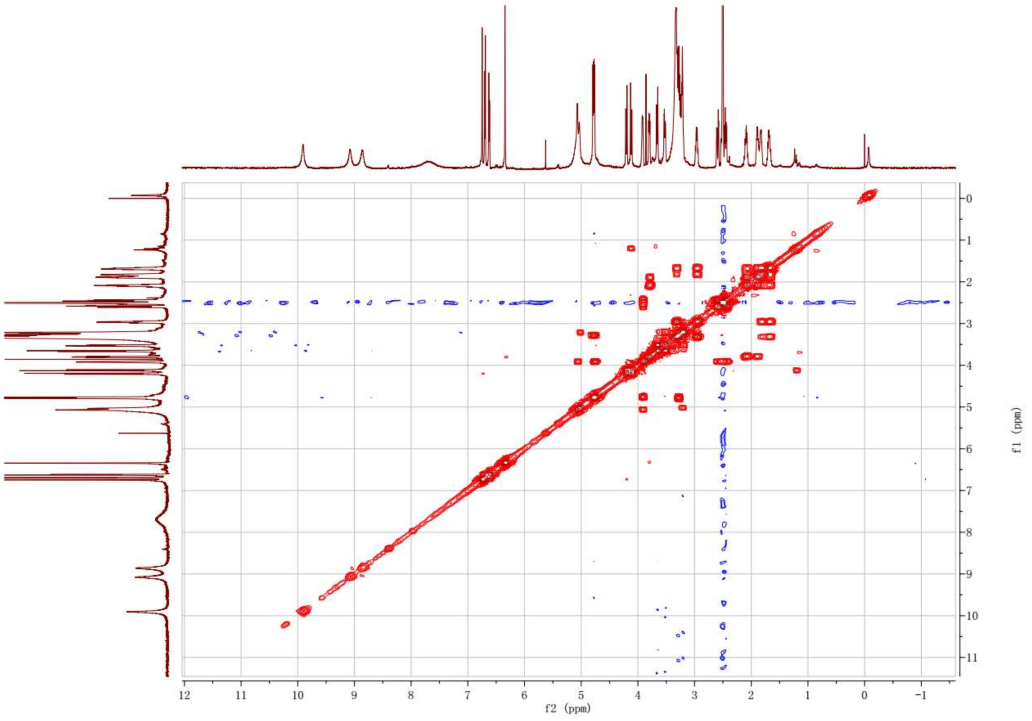

Figure S9. ^{1}H-^{1}H COSY spectrum of **1** inDMSO-d_6

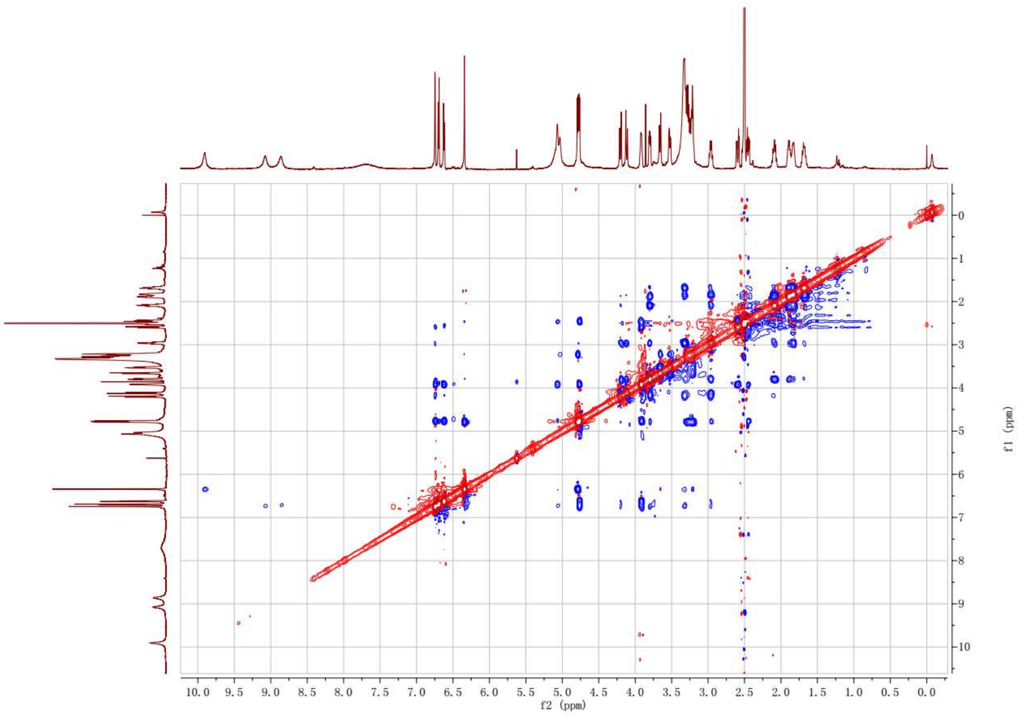

Figure S10. ROESY spectrum of **1** in DMSO-d_6

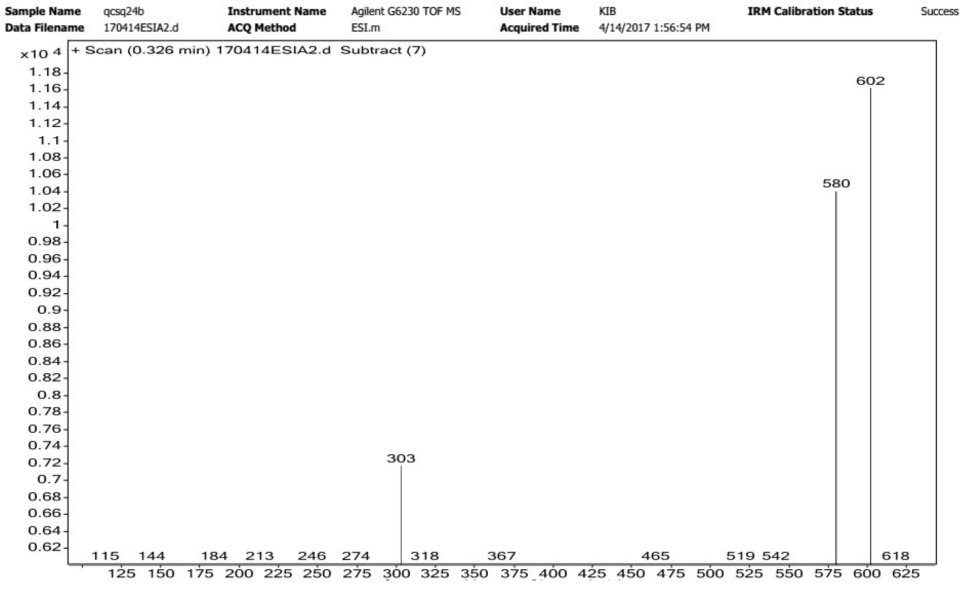

Figure S11. ESIMS of **1**

Qualitative Analysis Report

**Data Filename**	170414ESIA2.d	**Sample Name**	qcsq24b
**Sample Type**	Sample	**Position**	
**Instrument Name**	Agilent G6230 TOF MS	**User Name**	KIB
**Acq Method**	ESI.m	**Acquired Time**	4/14/2017 1:56:54 PM
**IRM Calibration Status**	Success	**DA Method**	ESI.m
**Comment**			

Sample Group **Info.**

Acquisition SW 6200 series TOF/6500 series
Version Q-TOF B.05.01 (B5125.2)

User Spectra

Fragmentor Voltage	Collision Energy	Ionization Mode
200	0	ESI

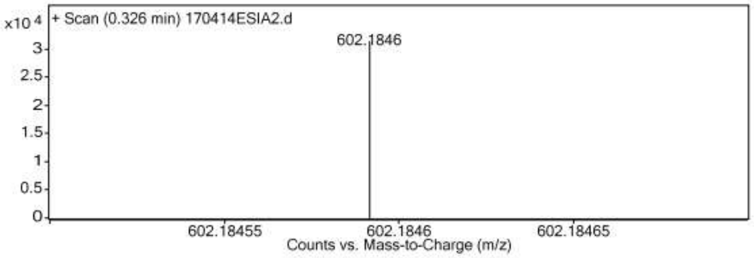

Peak List

m/z	z	Abund	Formula	Ion
121.0509		17087.65		
122.5475	2	15280.45		
143.0606	2	17177.86		
144.0598	2	9992.36		
274.274	1	26189.18		
303.086	1	11699.11		
318.3003	1	22738.51		
580.2022	1	17918.62		
602.1846	1	31253.29	C27 H33 N Na O13	M+
922.0098	1	16979.9		

Formula Calculator Element Limits

Element	Min	Max
C	0	200
H	0	400
O	9	15
Na	1	1
N	1	1

Formula Calculator Results

Formula	CalculatedMass	Mz	Diff.(mDa)	Diff. (ppm)	DBE
C27 H33 N Na O13	602.1850	602.1846	0.4	0.6	11.5

--- End Of Report ---

Figure S12. HRESIMS of **1**

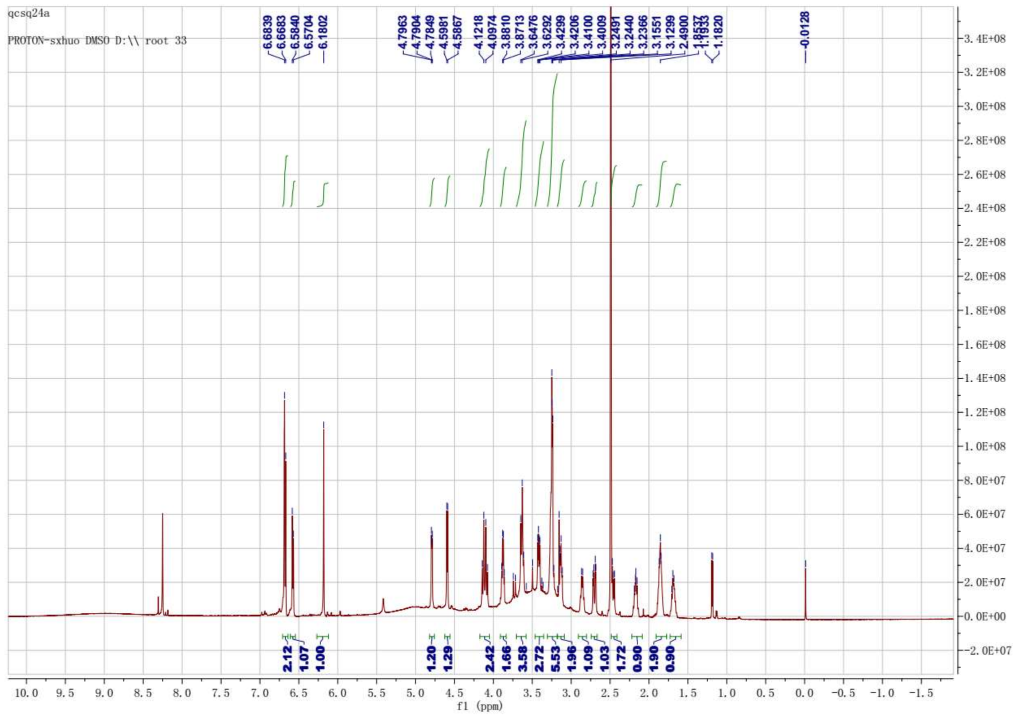

Figure S13. ^{1}H NMR spectrum of **2** in DMSO-d_6

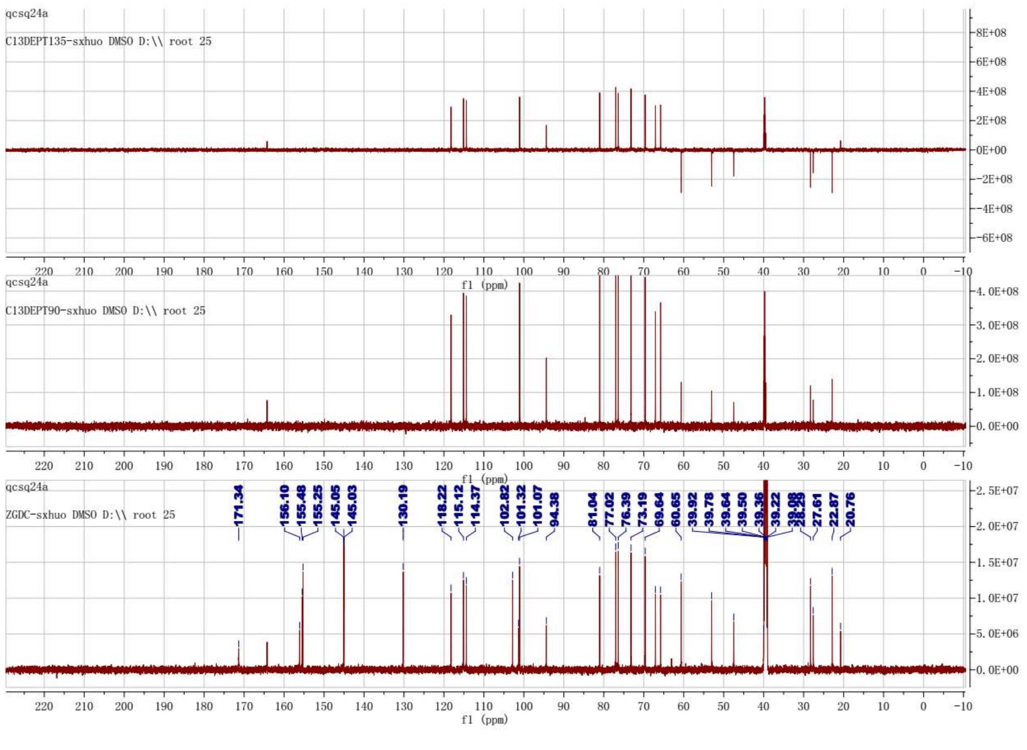

Figure S14. ^{13}C NMR and DEPT spectra of **2** in DMSO-d_6

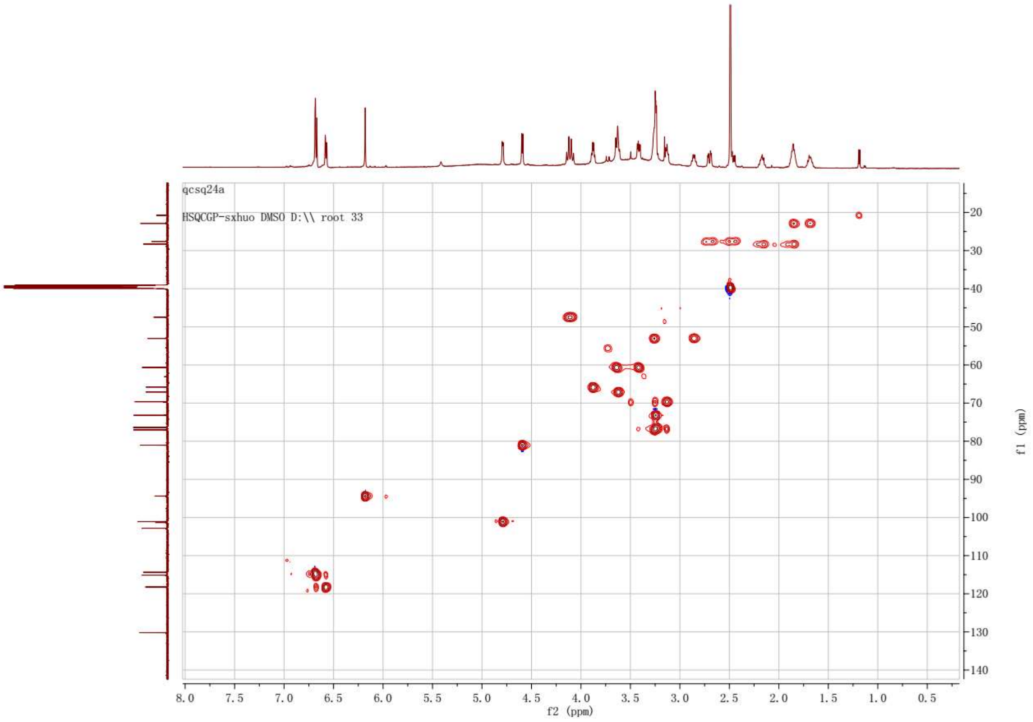

Figure S15. HSQC spectrum of **2** in DMSO-d_6

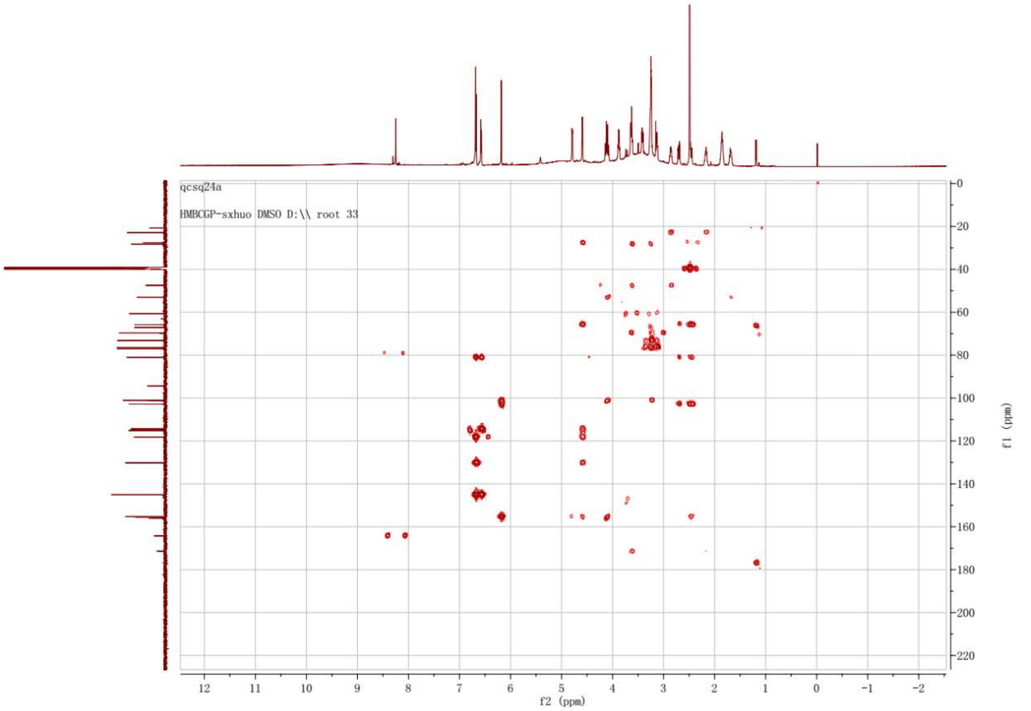

Figure S16. HMBC spectrum of **2** in DMSO-d_6

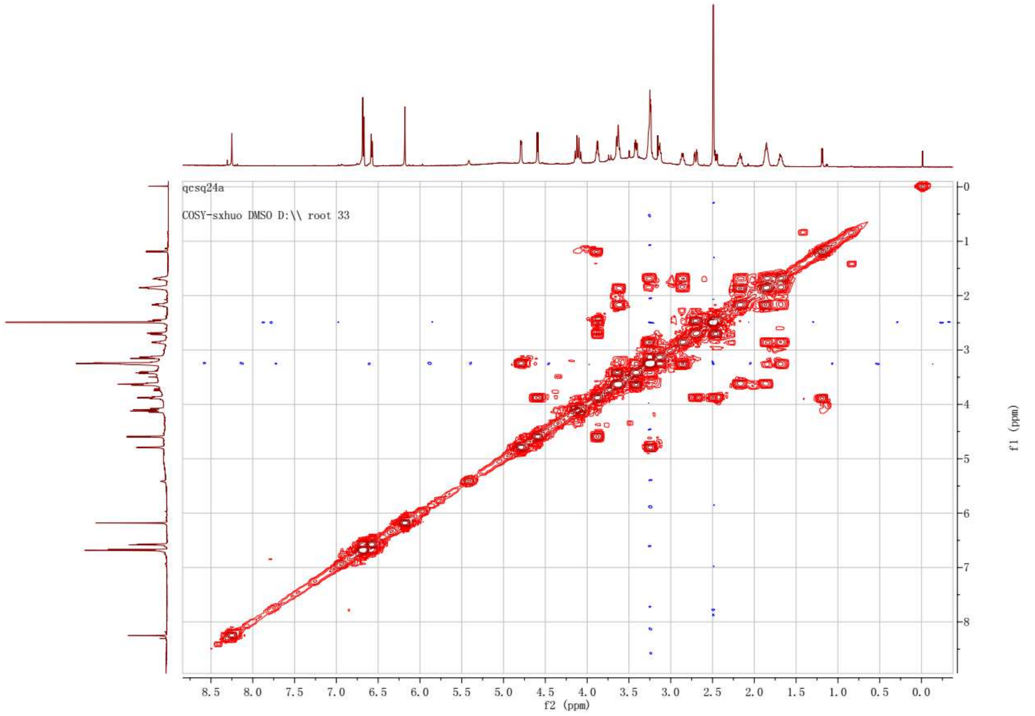

Figure S17. ^{1}H-^{1}H COSY spectrum of **2** in DMSO-d_6

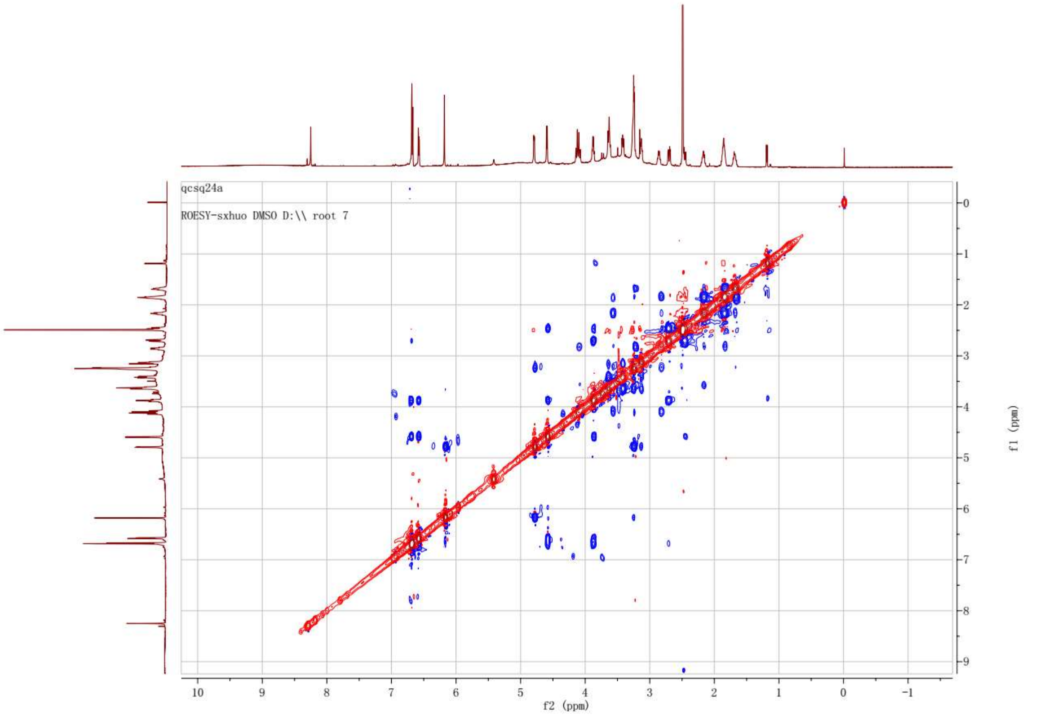

Figure S18. ROESY spectrum of **2** in DMSO-d_6

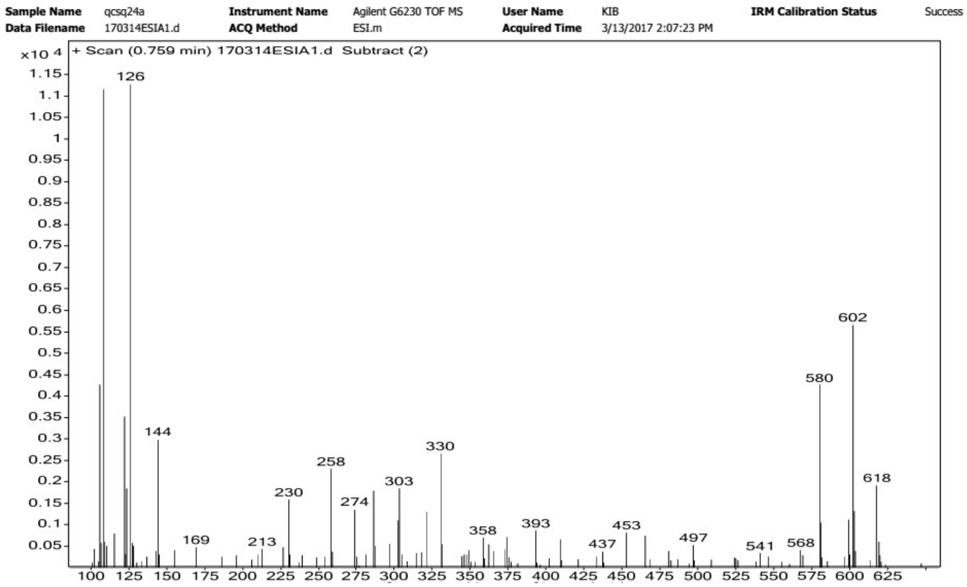

Figure S19. ESIMS of **2**

Qualitative Analysis Report

**Data Filename**	170314ESIA1.d	**Sample Name**	qcsq24a
**Sample Type**	Sample	**Position**	
**Instrument Name**	Agilent G6230 TOF MS	**User Name**	KIB
**Acq Method**	ESI.m	**Acquired Time**	3/13/2017 2:07:23 PM
**IRM Calibration Status**	Success	**DA Method**	ESI.m
**Comment**			

Sample Group **Info.**

Acquisition SW Version 6200 series TOF/6500 series Q-TOF B.05.01 (B5125.2)

User Spectra

Fragmentor Voltage	Collision Energy	Ionization Mode
200	0	ESI

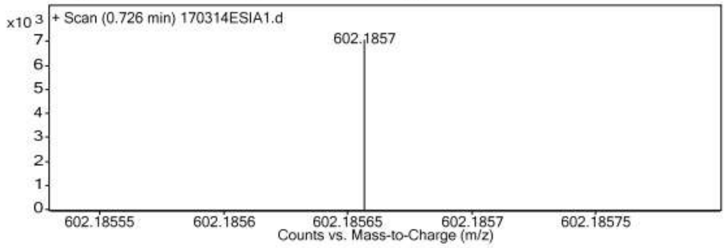

Peak List

m/z	z	Abund	Formula	Ion
121.0509		6373.15		
258.2793	1	4615.45		
286.3106	1	4008.49		
330.3375	1	6854.11		
349.1842	1	4156.34		
368.4257	1	8757.31		
393.2099	1	5436.61		
437.2344	1	4927.79		
481.2624	1	3878.74		
602.1857	1	7046.48	C27 H33 N Na O13	M+

Formula Calculator Element Limits

Element	Min	Max
C	0	200
H	0	400
O	9	16
Na	1	1
N	1	1

Formula Calculator Results

Formula	CalculatedMass	Mz	Diff.(mDa)	Diff. (ppm)	DBE
C27 H33 N Na O13	602.1850	602.1857	-0.7	1.2	11.5

--- End Of Report ---

Figure S20. HRESIMS of **2**
